# Ground Water Awareness Week — March 9–15, 2014

**Published:** 2014-03-07

CDC is collaborating with the National Ground Water Association to highlight National Ground Water Awareness Week, March 9–15, 2014. Many persons are not aware that much of the water they use flows from below ground to the surface to public water systems and private wells. The National Ground Water Association uses this week to stress the importance of ground water to the health and well-being of humans and the environment (1).

The majority of public water systems in the United States use ground water as their primary source, providing drinking water to almost 90 million persons (2). An additional 13 million U.S. homes use private wells, which also rely on ground water (3).

Usually, ground water in the United States is safe to use. However, ground water sources can be contaminated naturally or with pesticides, factory waste, and sewage as a result of imperfect agricultural, manufacturing, resource extraction, or sewage disposal practices of businesses or homes. The presence of contaminants at sufficient doses can lead to acute and chronic illness (4,5).

The U.S. Environmental Protection Agency has worked with individual states to develop new regulations to provide increased protection against microbial pathogens in public water systems that use ground water sources (6). Private ground water wells (i.e., those serving fewer than 25 persons) might not be regulated but nonetheless must be properly maintained by well owners to ensure that the water remains free from harmful chemicals and pathogens.* Resources are available from state and local health departments and nonprofit organizations to help homeowners protect their ground water.†

## Footnotes

* Additional information available at http://www.cdc.gov/healthywater/drinking/private/wells/index.html.

† Additional information available at http://www.apha.org/about/public+health+links/linksstateandlocalhealthdepartments.htm and http://www.cdc.gov/healthywater/drinking/training-education.html#webtraining.
